# Genome-Based Comparative Analyses of Antarctic and Temperate Species of *Paenibacillus*

**DOI:** 10.1371/journal.pone.0108009

**Published:** 2014-10-06

**Authors:** Melissa Dsouza, Michael W. Taylor, Susan J. Turner, Jackie Aislabie

**Affiliations:** 1 Centre for Microbial Innovation, School of Biological Sciences, University of Auckland, Auckland, New Zealand; 2 BioDiscovery New Zealand Limited, Parnell, Auckland, New Zealand; 3 Landcare Research, Hamilton, New Zealand; The University of Hong Kong, Hong Kong

## Abstract

Antarctic soils represent a unique environment characterised by extremes of temperature, salinity, elevated UV radiation, low nutrient and low water content. Despite the harshness of this environment, members of 15 bacterial phyla have been identified in soils of the Ross Sea Region (RSR). However, the survival mechanisms and ecological roles of these phyla are largely unknown. The aim of this study was to investigate whether strains of *Paenibacillus darwinianus* owe their resilience to substantial genomic changes. For this, genome-based comparative analyses were performed on three *P. darwinianus* strains, isolated from gamma-irradiated RSR soils, together with nine temperate, soil-dwelling *Paenibacillus* spp. The genome of each strain was sequenced to over 1,000-fold coverage, then assembled into contigs totalling approximately 3 Mbp per genome. Based on the occurrence of essential, single-copy genes, genome completeness was estimated at approximately 88%. Genome analysis revealed between 3,043–3,091 protein-coding sequences (CDSs), primarily associated with two-component systems, sigma factors, transporters, sporulation and genes induced by cold-shock, oxidative and osmotic stresses. These comparative analyses provide an insight into the metabolic potential of *P. darwinianus*, revealing potential adaptive mechanisms for survival in Antarctic soils. However, a large proportion of these mechanisms were also identified in temperate *Paenibacillus* spp., suggesting that these mechanisms are beneficial for growth and survival in a range of soil environments. These analyses have also revealed that the *P. darwinianus* genomes contain significantly fewer CDSs and have a lower paralogous content. Notwithstanding the incompleteness of the assemblies, the large differences in genome sizes, determined by the number of genes in paralogous clusters and the CDS content, are indicative of genome content scaling. Finally, these sequences are a resource for further investigations into the expression of physiological attributes that enable survival under extreme conditions and selection processes that affect prokaryotic genome evolution.

## Introduction

The phylum *Firmicutes* represents one of the most abundant and ubiquitous bacterial groups in the environment. Members of this phylum have been identified in a wide variety of habitats that vary in physical and biochemical characteristics, including the vertebrate gut [1], activated sludge [2], soil [3], [4], sediment [5], ocean waters [6], and lakewater [7]. Within soil, *Firmicutes* typically form a minor, yet consistent, component of microbial communities [8]–[11]. In addition, members of *Firmicutes* can be locally abundant, as observed in grassland soils of The Netherlands [12], and significantly more abundant in compacted forest [13] and arid soils [14] as compared to unimpacted, control soils. Within *Firmicutes*, members of the genera *Bacillus*, *Clostridium*, and *Paenibacillus* are commonly identified in soil microbial communities [15].

The genus *Paenibacillus* contains a monophyletic lineage of endospore-forming bacteria represented by over 100 described species [16]. Members of this genus have largely been isolated from terrestrial environments including cold soils of the Antarctic Peninsula [17], the Transantarctic Mountains, the Kafni glacier, Himalayas [18], Alaska [19] and from temperate soil environments, particularly those rich in humus and plant material [20], [21]. Their ability to successfully colonise these environments can be attributed to common physiological traits including formation of stress-resistant endospores, secretion of extracellular enzymes and anti-microbial compounds (that suppress growth of competing microorganisms), and the ability to hydrolyse a variety of carbohydrates including cellulose, starch, and xylan. They are also noted for their ability to form complex colonial patterns illustrating complex multicellular-like behaviours and for the production of phytohormones, antifungal compounds and nutrients including nitrate that promote plant health [16]. Whole genome sequencing can provide an insight into the molecular mechanisms of these physiological attributes and, more generally, elucidate the metabolic potential and ecological role of these species. To date, 67 *Paenibacillus* genomes have been sequenced, of which two, those of *P. larvae* [22], [23] and *P. vortex* [24], are accompanied by publications [25]. *P. larvae* was investigated for its ability to cause American foulbrood, a disease of honey bees, and *P. vortex* for its social organisation and complex pattern-forming behaviours.

Here, we report genome analyses of three *P. darwinianus* strains, isolated from gamma-irradiated soils of the Ross Sea Region (RSR), Antarctica [26]. Soils of the RSR represent a unique environment characterised by physical extremes of low temperature, elevated ultraviolet radiation and geochemical extremes of high salinity, low water and low nutrient availability [27]. Despite the harshness of this environment, many bacterial species prevail, with 16S rRNA gene pyrosequencing- and clone library-based studies identifying members of 15 bacterial phyla, namely *Acidobacteria*, *Actinobacteria*, *Armatimonadetes*, *Bacteroidetes*, *Chloroflexi*, *Cyanobacteria*, *Deinococcus*-*Thermus*, *Firmicutes*, *Gemmatimonadetes*, *Nitrospira*, *Planctomycetes*, *Proteobacteria*, *Spirochaetes*, *Verrucomicrobia* and Candidate ‘TM7’ [28]–[35]. Of these, *Firmicutes* were identified in most soil microbial communities, but their relative abundance was variable, and they typically formed a minor component of the community. However, they can be abundant, as observed in ornithogenic soils of Cape Hallett [36] and in mineral soils of the Wright Valley [31]. In addition, a pyrosequencing-based diversity study of airborne bacteria over Miers Valley identified members of the family *Paenibacillaceae* (of phylum *Firmicutes*) amongst the most abundant OTUs (operational taxonomic units) [37].

Members of the genus *Paenibacillus* are commonly cultured and identified in polar and permafrost soil communities. Despite this, only six novel *Paenibacillus* spp., namely *P. antarcticus* [38], *P. cineris*, *P. cookii* [39], *P. darwinianus* [26], *P. macquariensis* [40], and *P. wynnii* [17] have been isolated from the Antarctic environment, and little is known about the potential functions, or the metabolic contributions, of these bacteria in the Antarctic environment. A key question is whether strains of *P. darwinianus* owe their resilience to substantial genomic changes compared to *Paenibacillus* spp. isolated from temperate soil environments. Therefore, the objective of this study was to compare genomes of three strains of *P. darwinianus* with nine temperate, soil-dwelling *Paenibacillus* spp., focusing on traits that may contribute to survival and growth in soil, including signal transduction pathways, sigma factors, sporulation, motility and their ability to cope with oxidative and osmotic stress. This study has demonstrated that, while the genomic content investigated is largely similar across all 12 *Paenibacillus* isolates, there is some evidence for genome content scaling.

## Results and Discussion

### Genome overview

**Figure 1:**
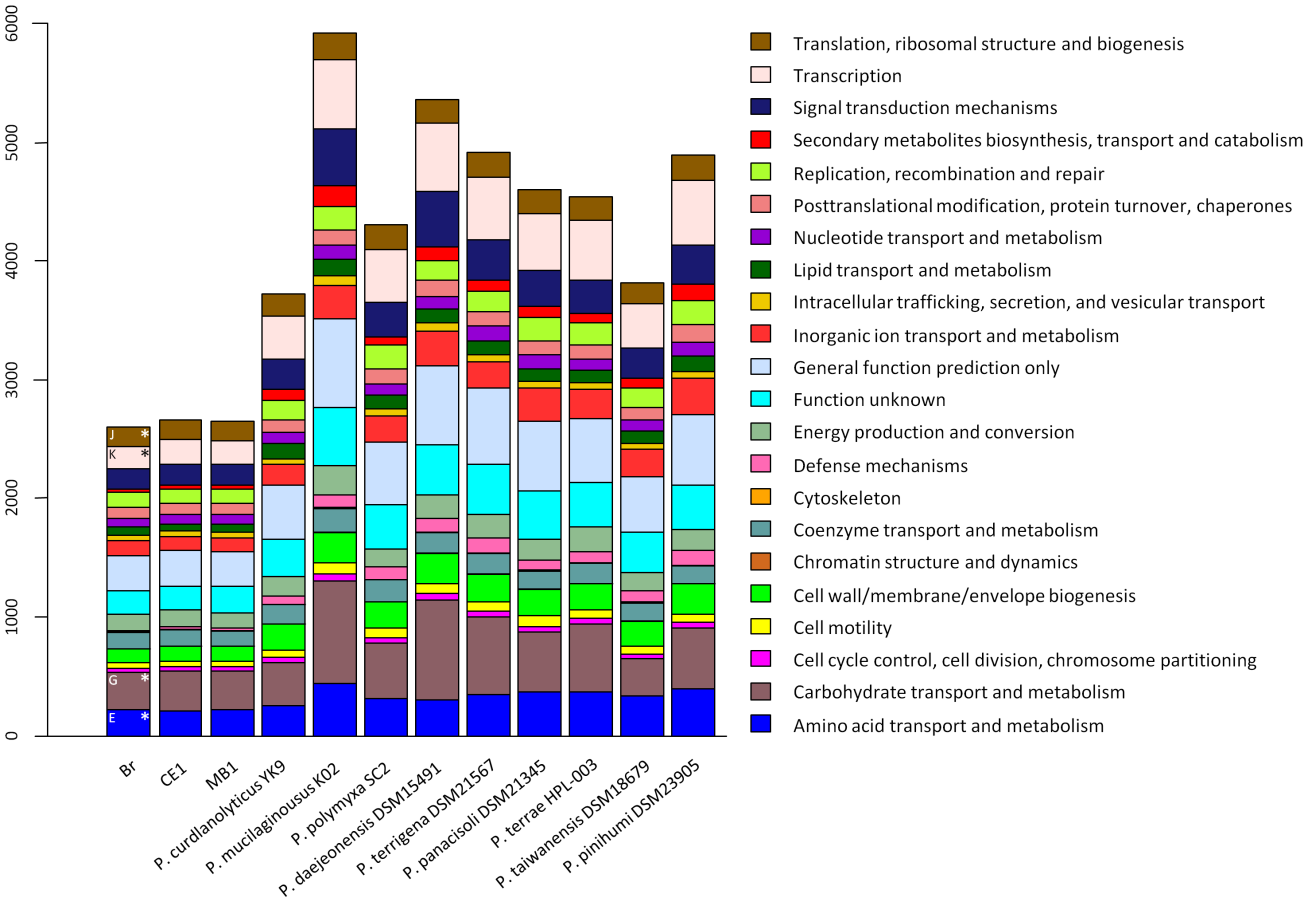
Comparison of gene content in temperate *Paenibacillus* spp. and *P. darwinianus* strains by COG categories.

The three *P. darwinianus* genomes are composed of the chromosome (approx. 3 Mbp), each containing a varying number of DNA scaffolds ranging from 107 to 111. The genome completeness was approx. 88%, assessed by the occurrence of essential, single-copy genes. Due to this incompleteness, we have regarded the apparent absence or low copy number of a given gene with caution. General genome features of the *P. darwinianus* strains compared with nine temperate *Paenibacillus* spp. are listed in Table 1. Dot plot diagrams comparing levels of homology between the three *P. darwinianus* genomes were generated on the IMG website. These plots revealed high levels of synteny between the three strains (Figure S1). The genome G+C content for the three strains was approx. 56%, in accordance with other members of *Paenibacillus*, yet significantly different to that of the nine temperate species (P<0.01). The genomes contained 3,101–3,123 open reading frames with an average coding density of 87.4%. These genomes comprised approx. 3,000 protein coding sequences (CDSs). For each genome, approx. 77% of the total CDSs were assigned to clusters of orthologous groups (COG) categories. Notably, the highest proportion of genes was assigned to COG categories including amino acid transport and metabolism [E], carbohydrate transport and metabolism [G], transcription [K], and translation, ribosomal structure and biogenesis [J] (Figure 1). Transmembrane proteins constituted approx. 25% of the total CDSs identified. Putative horizontally transferred genes constituted 1.87–2.29% of the total genes observed. Significantly fewer copies of the 16S rRNA gene were identified in *P. darwinianus* genomes as compared to the temperate *Paenibacillus* spp. (P<0.01). Temperature range data indicated that *P. darwinianus* strains are psychrotolerant with optimal, minimal and maximal growth occurring at 18, 10 and 37°C, respectively [26]. The temperate *Paenibacillus* spp. exhibit growth over a wide range of temperatures (10–45°C) with optimal growth occurring between 25 and 30°C [16], [20], [41], [42].

**Table 1:** General genome features of *P. darwinianus* genomes (this study) vs. nine temperate, soil-dwelling *Paenibacillus* spp. [100].

	1	2	3	4	5	6	7	8	9	10	11	12
**Genome data**												
Genome size (bp)	3,021,550	3,056,161	3,051,566	5,452,778	8,770,140	6,241,931	7,464,058	6,361,561	6,326,414	6,083,395	5,247,653	6,760,575
DNA coding region (%)	2,645,264 (87.55)	2,672,123 (87.43)	2,663,886 (87.30)	4,835,968 (88.69)	7,182,175 (81.89)	5,308,414 (85.04)	6,619,323 (88.68)	5,583,508 (87.77)	5,389,440 (85.19)	5,167,317 (84.94)	4,508,951 (85.92)	5,851,762 (86.56)
G+C content (%)	56.23	56.14	56.08	51.94	58.24	44.62	53.22	46.08	48.22	46.77	44.79	48.53
Scaffold count	111	108	107	40	1	2	36	40	43	1	20	45
Total RNA genes	58	53	72	133	224	196	103	118	127	117	100	105
tRNA genes	23	21	34	101	189	14	61	71	87	89	68	64
16S rRNA genes	4	4	5	10	13	14	7	10	7	9	8	8
Other RNA genes	29	24	26	-	-	-	29	27	26	-	16	26
Total number of genes	3,101	3,123	3,163	4,957	7,476	6,228	6,575	5,932	5,718	5,642	4,779	6,169
Total protein CDSs (%)	3,043 (98.13)	3,070 (98.30)	3,091 (97.72)	4,824 (97.32)	7,252 (97)	6,032 (96.85)	6,472 (98.43)	5,814 (98.01)	5,591 (97.78)	5,525 (97.93)	4,679 (97.91)	6,604 (98.30)
With function prediction (%)	2,487 (80.20)	2,540 (81.33)	2,535 (80.15)	3,488 (70.37)	4,389 (58.71)	4,463 (71.66)	5,199 (79.07)	4,777 (80.53)	4,389 (76.76)	3,768 (66.78)	3,692 (77.52)	4,754 (77.06)
Without function prediction (%)	556 (17.93)	530 (16.97)	556 (17.58)	1,336 (26.95)	2,863 (38.30)	1,569 (25.19)	1,273 (19.36)	1,037 (17.48)	1,202 (21.02)	1,757 (31.14)	987 (20.65)	1,310 (21.24)
With COGs (%)	2,389 (77.04)	2,431 (77.84)	2,426 (76.70)	3,390 (68.39)	5,362 (71.72)	3,906 (62.72)	4,887 (74.33)	4,468 (75.32)	4,151 (72.60)	4,117 (72.97)	3,461 (72.42)	4,443 (72.02)
Coding for transmembrane proteins (%)	71 (2.29)	59 (1.89)	59 (1.87)	398 (8.03)	-	257 (4.13)	302 (4.59)	240 (4.05)	99 (1.73)	-	101 (2.11)	283 (4.59)
Genome Completeness (%)	87	88	88	-	-	-	-	-	-	-	-	-
**Metadata**												
Isolation source	Britannia, Darwin Mountains	Cape Evans, Ross Sea Region	Minna Bluff, Ross Sea Region	Kobe city, Japan	-	China	Daejeon, South Korea	Chiba, Japan	Pocheon Province, South Korea	Gara Mountains,	Wu-Feng, Taiwan	Daejeon, South Korea
Habitat	Gamma-irradiated soil	Gamma-irradiated soil	Gamma-irradiated soil	Soil	Soil	Rhizosphere soil	Soil	Soil	Soil	Soil	Farmland soil	Rhizosphere soil
Temperature range (°C)	10–37	10–37	10–37	30**	10–45*	30**	30**	4–32#	15–45*	10–40*	10–45$	15–37%

1, *P. darwinianus* Br; 2, *P. darwinianus* CE1; 3, *P. darwinianus* MB1; 4, *P. curdlanolyticus* YK9; 5, *P. mucilaginous* K02; 6, *P. polymyxa* SC2; 7, *P. daejeonensis* DSM 15491; 8, *P. terrigena* DSM 21567; 9, *P. panacisoli* DSM 21345; 10, *P. terrae* HPL-003; 11, *P. taiwanensis* DSM 18679; 12, *P. pinihumi* DSM 23905.

*, Data from [16];

**, Optimal temperature reported [16];

# , Data from [41];

^$^, Data from [42];

% , Data from [20].

### General genome comparisons

**Figure 2:**
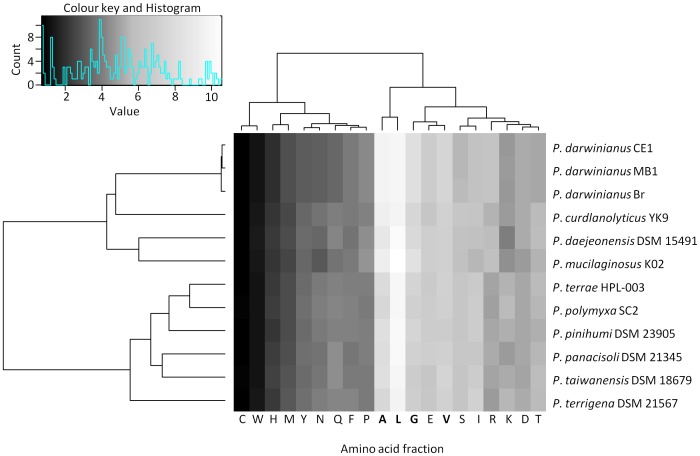
Amino acid usage heatmap of temperate *Paenibacillus* spp. and *P. darwinianus* strains.

**Figure 3:**
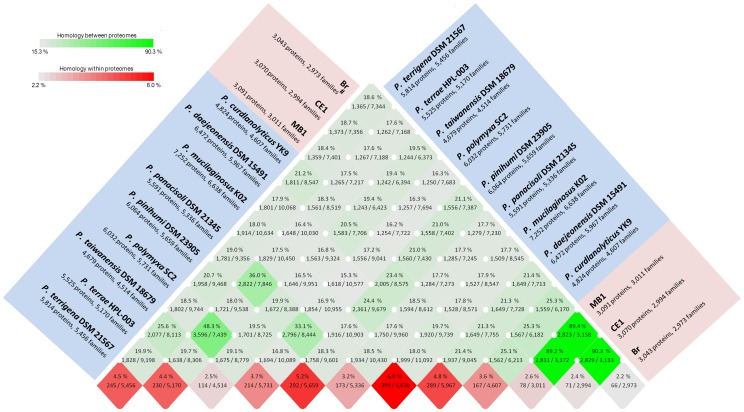
A BLAST matrix of an all against all protein comparison of 12 *Paenibacillus* genomes.

General comparisons between genomes of the three *P. darwinianus* strains and nine temperate *Paenibacillus* spp. were carried out using CMG-Biotools [43]. Firstly, the amino acid composition of protein sequences from the 12 *Paenibacillus* spp. was determined. This analysis revealed broad similarities in genome-wide amino acid usage profiles for all 12 *Paenibacillus* spp. with Ala, Leu, Gly, and Val the most frequently used amino acids across all genomes (Figure 2). Predicted proteome comparisons and a pan- and core-genome plot analysis were also performed on all 12 *Paenibacillus* genomes using CMG-Biotools [43]. Proteomes were predicted using Prodigal [44] and then BLAST algorithm (Basic Local Alignment Search Tool)-based proteome comparisons were performed to identify whether proteins are shared between predicted proteomes [45]. The main part of the matrix (shaded green) (Figure 3) consists of pairwise proteome comparisons and the bottom row (shaded red) represents a self-comparison where a hit within the proteome to a protein other than the query is identified as an internal homolog or a paralog. The BLAST matrix illustrates that the conservation between genomes is higher within species than within a genus. *P. darwinianus* strains exhibited a high level of conservation of gene families (89.2–90.3%). This observation was also supported by the pan- and core-genome analysis, as the *P. darwinianus* core- and pan-genome comprised 2,735 and 3,194 gene families, respectively. A large proportion of genes in the *P. darwinianus* core-genome were assigned to COG categories including carbohydrate transport and metabolism [G], amino acid transport and metabolism [E], and transcription [K]. In contrast, the temperate *Paenibacillus* core- and pan-genome comprised 1,139 and 25,493 gene families, respectively. Genes in the temperate *Paenibacillus* core-genome were assigned to COG categories including translation, ribosomal structure and biogenesis [J], amino acid transport and metabolism [E], and transcription [K]. Overall, the temperate and Antarctic core- and pan-genome comprised 998 and 26,612 gene families, respectively. Finally, up to 6% of the CDSs were in paralogous clusters for temperate *Paenibacillus* spp., whereas approx. 2.4% of the CDSs were in paralogous clusters for all *P. darwinianus* strains (Figure 3).

### Genomic features linked to survival in stressful conditions

#### Two-component signal transduction systems

Two-component signal transduction systems (TCS) represent a primary means by which a bacterial cell senses and responds to a variety of stresses and to the changing environment. *P. darwinianus* genomes contained 78–82 genes associated with TCS including approx. 40 genes for membrane-bound histidine kinases and 41 for response regulators (Table S1). A large number of genes for TCS were also identified in the psychrophilic archaeon *Methanococcoides burtonii* [46] and bacterium *Desulfotalea psychrophila* [47], further illustrating the importance of signal transduction systems for growth and survival in cold environments. In *P. darwinianus* genomes, approx. 2.6% of the total CDSs can be attributed to TCS. A similar proportion of genes (approx. 3% of the total CDSs) were attributed to TCS in genomes of the temperate *Paenibacillus* species. Genes for TCS, DesK-DesR for low temperature [48], CheA-CheY for chemotaxis [49], PhoR-PhoP for phosphate regulation [50], ResE-ResD for oxygen limitation [51], DegS-DegU for exoprotease production, competence development, biofilm, flagellum and complex colony formation [52], were identified in all 12 *Paenibacillus* genomes (Table S2). Notably, genes for TCS, LytS-LytR (for the regulation of autolysis), were identified in all *P. darwinianus* genomes and in the genome of just one temperate *Paenibacillus* species, *P. mucilaginosus*. However, the *lrgAB* operon, induced by the *lytSR* operon and responsible for blocking the activity of murein hydrolases (enzymes that have the ability to degrade bacterial cell wall), was not identified in *P. darwinianus* genomes [53]. In the psychrophilic bacterium, *Flavobacterium psychrophilum*, expression of the sensor kinase LytS was cold-induced, with expression significantly upregulated at 8°C compared to its expression at 20°C [54]. In the Antarctic soil environment, regulated autolysis may allow for the recycling of cellular components, thus providing bacterial communities with nutrients.

With the exception of ornithogenic soils formed under penguin rookeries, RSR soils typically contain low concentrations of organic carbon, ranging from 0.01 to 0.96 mg C g^−1^ soil [55]. Therefore the ability to detect labile C sources such as dicarboxylic acids can be crucial for survival in the Antarctic soil environment. Organic compounds including C_4_-dicarboxylates, oxalate and succinate have been identified in aerosol particles over coastal East Antarctica [56], Showa Station [57] and Finnish Station Aboa [58] in Queen Maud Land. Genes for TCS, DctS-DctR (for the detection of aerobic C_4_-dicarboxylates, namely succinate, fumarate, malate, and oxaloacetate) were identified in the *P. darwinianus* genomes and in the genomes of just two temperate *Paenibacillus* spp., *P. mucilaginosus* and *P. terrae*. Genes encoding additional proteins required for the function of sensor kinase, DctS including DctA and DctB, were also identified in all *P. darwinianus* genomes [59].

#### Sigma factors

Sigma factors are dissociable units of RNA polymerase that activate the conditional expression of a specific set of genes in response to a particular stress or stimulus, thus implementing compensatory physiological changes. *P. darwinianus* genomes contained genes associated with a diverse set of sigma factors similar to the multiple copies observed in psychrophilic bacteria *Planococcus halocryophilus* [60] and *Psychromonas ingrahamii* [61] (Table S3). These included the primary housekeeping factor, σ^A^ and alternative sigma factors including general stress response factor, σ^B^, chemotaxis and flagellar motility associated factor, σ^D^, cell envelope stress associated factor, σ^ECF^, cold shock response factor, σ^L^, and sporulation-specific factors, σ^E^ σ^F^, σ^G^, and σ^H^ [62], [63]. Among these factors, σ^B^ is crucial as it controls the expression of an estimated 150 or more genes in response to a wide range of stress and starvation conditions [64]. Common regulators of σ^B^, including RsbW and RsbV, were also identified in all *P. darwinianus* strains.

#### Oxidative and osmotic stress resistance

Reactive oxygen species (ROS) including superoxides (O_2_^−^), hydrogen peroxide (H_2_O_2_), hydroxyl radical molecules (⋅OH) and lipid peroxides, are both produced and accumulated as a result of aerobic metabolism [65]. Additionally, Antarctic soil bacteria are exposed to low temperatures that increase ROS stability and improve oxygen solubility [66]. Consequently, combating free radical damage is essential for survival in the Antarctic soil environment. Genomes of all three *P. darwinianus* strains contained two copies of the catalase gene, two of the superoxide dismutase gene *sodA* and one of the DNA oxidative damage protectant gene *dps*. Additionally, up to six copies of the peroxiredoxin genes, *ahpCF*, *bcp* and *tpx* and nine copies of the thioredoxin genes, *trxA* and *trxB* were identified in all *P. darwinianus* strains (Table 2, Table S10). Other bacteria that also contain multiple copies of genes for ROS detoxification include *Colwellia psychrerythraea* [67], *D. psychrophila* [47], and *P. halocryophilus* [60]. These genes were also identified in genomes of all temperate *Paenibacillus* spp. In *Bacillus subtilis*, the oxidative stress response is regulated by proteins, PerR and OhrR, activated by peroxides and by proteins, σ^B^ and Spx, that are in turn activated under diverse stress conditions [68], [69]. Genes for regulatory proteins PerR, OhrR and σ^B^ were identified in all *P. darwinianus* genomes. However, Spx was only identified in *P. darwinianus* strain CE1. In genomes of the temperate *Paenibacillus* spp., these regulatory proteins were largely absent.

**Table 2:** Oxidative and osmotic stress response associated genes in genomes of temperate *Paenibacillus* spp. and *P. darwinianus* strains.

Product name	Gene Symbol	Enzymes	COGs	Pfams	1	2	3	4	5	6	7	8	9	10	11	12
***Oxidative stress response***																
Catalase	*katE*	EC:1.11.1.6	COG0753	pfam00199, pfam06628	1	1	1	3	3	1	4	2	1	1	-	1
Mn-containing catalase	*-*	*-*	COG3546	pfam05067	1	1	1	1	3	3	3	1	2	-	2	2
Superoxide dismutase	*sodA*	EC:1.15.1.1	COG0605	pfam00081 pfam02777	2	2	2	2	1	2	2	2	2	2	3	2
DNA-binding ferritin-like protein	*dps*	*-*	COG0783	pfam00210	1	1	1	1	4	1	1	1	1	1	1	2
Peroxiredoxin	*ahpCF*	*-*	COG0450	pfam00578	1	1	1	-	-	1	1	1	1	1	2	2
Peroxiredoxin	*tpx*	EC:1.11.1.15	COG2077	pfam08534	1	2	1	-	-	1	1	1	1	1	1	1
Peroxiredoxin	*bcp*	EC:1.11.1.15	COG1225	pfam00578, pfam08534	3	3	4	-	-	1	4	3	1	1	3	8
Thioredoxin/Thioredoxin domain-containing protein	*trxA*	*-*	COG0526, COG3118, COG0694	pfam00085	5	5	5	3	6	6	6	7	6	2	5	4
Thioredoxin reductase	*trxB*	EC:1.8.1.9	COG0492	pfam13738	5	5	5	2	-	3	10	6	5	5	5	8
Fe2+/Zn2+ uptake regulation proteins	*perR, fur*	*-*	COG0735	pfam01475	1	1	1	1	1	-	-	-	-	-	-	-
OhrR-like transcriptional regulator	*ohrR, marR*	*-*	COG1846	pfam01047	1	1	1	-	-	1	-	-	-	1	-	-
Transcriptional regulator Spx	*spx, arsC*	-	COG1393	pfam03960	-	1	-	-	-	-	1	1	1	-	1	1
***Osmotic stress response***																
ABC-type proline/glycine betaine transport system	*opuA*	EC:3.6.3.32	COG4175, COG4176, COG2113	pfam00005, pfam00571, pfam00528, pfam04069	3	3	3	-	-	2	3	3	3	2	-	3
Sodium:solute symporter family	*opuE*	*-*	COG4147, COG0591	-	2	2	2	-	-	-	-	-	-	1	-	-
γ-glutamyl phosphate reductase	*proA*	EC:1.2.1.41	-	pfam39821	1	1	1	1	1	1	1	1	1	1	1	1
γ -glutamyl kinase	*proB*	EC:2.7.2.11	COG0263	pfam00696, pfam01472	1	1	1	1	1	1	1	1	1	-	1	1
Pyrroline-5-carboxylate reductase	*proC*	EC:1.5.1.2	COG0345	pfam03807, pfam14748	2	2	2	2	1	2	2	1	2	1	2	1

1, *P. darwinianus* Br; 2, *P. darwinianus* CE1; 3, *P. darwinianus* MB1; 4, *P. curdlanolyticus* YK9; 5, *P. mucilaginous* K02; 6, *P. polymyxa* SC2; 7, *P. daejeonensis* DSM 15491; 8, *P. terrigena* DSM 21567; 9, *P. panacisoli* DSM 21345; 10, *P. terrae* HPL-003; 11, *P. taiwanensis* DSM 18679; 12, *P. pinihumi* DSM 23905.

Numbers in each column represent copy numbers per genome. Locus tags for each copy number are listed in Table S10.

Salinity is a prominent feature of Antarctic soils, with some soils such as those in central Wright Valley containing water-soluble salts as high as 10 g cm^−2^ soil [70]. Accumulation of osmoprotectants such as glycine betaine and proline is an effective strategy to combat hyper-osmotic stress. Genes involved in glycine betaine uptake (*opuA*) comprising three components: OpuAA, the ATPase component, OpuAB, the permease component and OpuAC, the periplasmic component, were identified in all *P. darwinianus* strains and in most temperate *Paenibacillus* spp. [71]. Additionally, genes for high-affinity proline-specific uptake by the sodium:solute symporter, OpuE were identified in all *P. darwinianus* genomes and in the genome of just one temperate species, *P. terrae*. Proline biosynthesis enzymes, γ-glutamyl kinase (*proB*), γ-glutamyl phosphate reductase (*proA*) and Pyrroline-5-carboxylate reductase (*proC*) were also identified in all *Paenibacillus* genomes [72] (Table 2, Table S10). Multiple copies of genes for the uptake of these osmoprotectants were also identified in genomes of several cold-adapted bacteria including C. *psychrerythraea* [67], *P. halocryophilus* [60], *Pseudoalteromonas haloplanktis* [73], and *P. ingrahamii* [61]. Hypo-osmotic stress, a consequence of frequent freeze-thaw cycles, is also an important feature of the Antarctic soil environment. All *P. darwinianus* strains contained genes for mechanosensitive ion channels (MScL), responsible for the release of cytoplasmic solutes [74]. Genes for this transporter were also identified in genomes of all temperate *Paenibacillus* spp. (Table S4).

#### Transporter genes

Antarctic *Paenibacillus* genomes contained 411–422 membrane transport protein-related genes that constitute approx. 14% of the total CDSs (Table S4). Genomes of the temperate *Paenibacillus* spp. contained a similar proportion of membrane transport-related genes. Genes encoding ATP-binding cassette (ABC)-type transporters associated with amino acid, antimicrobial peptide, sugar, nitrate and proline/glycine betaine transport, comprised approx. 60% of the membrane transporter genes (Table S5). It is notable that significantly fewer copies of the ABC-type multidrug transport system were identified in *P. darwinianus* strains as compared to the temperate species (P<0.01). While temperate *Paenibacillus* spp. contained several copies of genes encoding ABC-type oligopeptide, ABC-type dipeptide/oligopeptide/nickel, and ABC-type polysaccharide/polyol phosphate transport systems, no copies of genes associated with these transport systems were identified in the *P. darwinianus* genomes. However, a similar proportion of CDSs were associated with carbohydrate metabolism across all *Paenibacillus* genomes (Table S6). Therefore, the limited catabolic activity demonstrated by *P. darwinianus* strain Br^T^ in Biolog's phenotype microarray-based assay [26], may be due to its inability to transport polysaccharides and peptides, suggestive of an adaptation to the nutrient-limited Antarctic soil environment.

#### Cold shock response

During summer, diurnal temperature fluctuations are common in Antarctic soils. During this period, air temperatures are often below 0°C, however surface soils can be heated up to 10–15°C at midday depending on the position of the sun and cloud cover, and drop to below 0°C overnight [75], [76]. Low temperatures have a major impact on the structure and function of cellular constituents including the membrane, the ribosome and nucleic acids by decreasing membrane fluidity, reducing ribosome function, unwinding the DNA double helix and by stabilizing secondary structures of nucleic acids, thus reducing mRNA transcription and translation [77], [78]. Genome analysis of *P. darwinianus* strains revealed the presence of 4–5 copies of genes for cold-shock proteins similar to the multiple copies found in C. *psychrerythraea* [67], *Psychrobacter arcticus* [79] and *Shewanella oneidensis* [80]. In addition, several genes for cold-shock induced proteins, comparable to those identified in the transcriptome of cold-shocked *B. subtilis* [81] were also identified in all *P. darwinianus* genomes (Table 3, Table S11). A large proportion of these genes were also identified in temperate *Paenibacillus* spp.

**Table 3:** Cold-shock induced genes identified in genomes of temperate *Paenibacillus* spp. and *P. darwinianus* strains.

Product name	Gene symbol	COGs	Pfams	1	2	3	4	5	6	7	8	9	10	11	12
***Adaptation to cold-shock***															
Cold shock proteins	*cspB, cspC*	COG1278	pfam00313	5	5	4	5	1	3	3	3	3	3	3	4
***Metabolism of lipids***															
Fatty acid desaturase	*desA*	COG3239	pfam00487	1	1	3	1	1	-	1	1	1	1	1	2
***Metabolism of amino acids***															
Leucine dehydrogenase	*bcd*	COG0334	pfam00208, pfam02812	3	3	3	-	-	-	2	1	-	-	1	1
2-oxoisovalerate dehydrogenase (E3 subunit, dihydrolipoamide dehydrogenase)	*lpd*	COG1249	pfam00070, pfam02852, pfam07992	3	4	3	2	-	-	4	4	3	-	3	3
2-oxoisovalerate dehydrogenase (E1 alpha subunit)	*bkdAA*	COG1071	pfam00676	1	1	1	1	1	1	1	1	1	1	1	1
2-oxoisovalerate dehydrogenase (E1 beta subunit)	*bkdAB*	COG0022	pfam02779, pfam02780	1	1	1	1	1	1	1	1	1	1	1	1
2-oxoisovalerate dehydrogenase (E2 subunit, lipoamide acyltransferase)	*bkdB*	COG0508	pfam00198, pfam00364, pfam02817	3	3	3	-	-	-	2	3	3	1	3	2
***Metabolism of nucleotides***															
Adenylosuccinate synthetase	*purA*	COG0104	pfam00709	-	-	1	2	1	1	2	2	1	1	2	2
***Metabolism of carbohydrates***															
Fructose-1,6-bisphosphate aldolase	*fbaA*	COG0191	pfam01116	1	1	1	1	2	1	2	1	1	1	1	2
***ABC-type Transporter***															
ABC-type Mn/Zn transport systems, binding protein	*mntA*	COG0803	pfam01297	1	1	1	1	2	2	2	2	3	2	2	4
ABC-type Mn/Zn transport systems, ATPase component	*mntB*	COG1121	pfam00005	1	1	1	1	2	2	2	2	3	2	3	5
ABC-type Mn/Zn transport systems, permease component	*mntC*	COG1108	pfam00950	1	1	1	1	2	3	2	3	4	3	4	5
***RNA synthesis - elongation***															
DNA-directed RNA polymerase, delta subunit	*rpoE*	COG3343	*-*	1	1	1	1	1	1	1	1	1	1	1	1
***Protein synthesis (ribosomal proteins)***															
30S ribosomal protein S15	*rpsO*	COG0184	pfam00312	1	1	1	1	1	1	1	1	1	1	1	1
50S ribosomal protein L27	*rpmA*	COG0211	pfam01016	1	1	1	1	-	1	1	1	1	1	1	1
50S ribosomal protein L31	*rpmE*	COG0254	pfam01197	1	1	1	1	1	2	1	1	1	2	1	2
50S ribosomal protein L32	*rpmF*	COG0333	pfam01783	1	1	1	1	1	1	1	1	1	1	1	1
***Unknown function***															
Cold-inducible protein YdjO	*ydjO*	-	pfam14169	1	1	-	-	-	-	1	1	2	-	2	-

1, *P. darwinianus* Br; 2, *P. darwinianus* CE1; 3, *P. darwinianus* MB1; 4, *P. curdlanolyticus* YK9; 5, *P. mucilaginous* K02; 6, *P. polymyxa* SC2; 7, *P. daejeonensis* DSM 15491; 8, *P. terrigena* DSM 21567; 9, *P. panacisoli* DSM 21345; 10, *P. terrae* HPL-003; 11, *P. taiwanensis* DSM 18679; 12, *P. pinihumi* DSM 23905.

Numbers in each column represent copy numbers per genome. Locus tags for each copy number are listed in Table S11.

#### Sporulation

Spores exhibit a high degree of resistance to various stresses including low temperatures, frequent freeze-thaw cycles, UV and gamma radiation, extreme desiccation, and low availability of nutrients that are all common features of the Antarctic soil environment. *P. darwinianus* genomes encode an extensive set of 63–78 genes (2.3% of the total CDSs) responsible for various facets of sporulation including DNA replication and translocation, formation of the sporulation septum, engulfment, spore morphogenesis and germination (Table S7). In comparison, genomes of temperate *Paenibacillus* spp. contained 94–112 genes (1.7% of the total CDSs) associated with sporulation (Table S8).

#### Motility and chemotaxis

In soil environments, active movement by bacteria towards regions that contain higher concentrations of beneficial compounds including water and nutrients may be crucial for survival. Over 40 genes are required for flagellar assembly and movement, including structural subunits for the synthesis of the basal body, the hook and the filament, regulatory proteins σ^D^, FlgM and CodY, motor force generators MotA, MotB and chemosensory proteins [82], [83]. Chemosensory proteins comprise four groups, firstly a signal recognition and transduction group containing methyl-accepting chemotaxis proteins and glutamine deamidase, CheD, secondly an excitation group containing histidine kinase, CheA, coupling protein, CheW, and response regulator, CheY, an adaptation group containing methyl transferase, CheR and methyl esterase, CheB and finally a signal removal group containing phosphatase, CheC [84]. While approx. 50 genes (1.6% of the total CDSs) encoding components of the flagellum and chemosensory pathways were identified in *P. darwinianus* genomes, temperate *Paenibacillus* genomes contained 63–98 genes (1.3% of the total CDSs) associated with flagella biosynthesis and chemosensory pathways (Table S9). In addition, cells of *P. darwinianus* Br are known to possess a monotrichous flagellum. However, no motility was observed in cells of strain Br, indicating that further investigation is necessary to identify the stimulus needed for movement [26]. A similar observation was made for cells of *P. ingrahamii* that showed no motility despite the presence of a large cluster of flagellar genes [61]. It is hypothesized that the lack of observed motility may be due to a lack of appropriate stimulus or a defect in one of the essential flagellar proteins or in the expression or assembly processes.

## Conclusions

Previous studies have shown that, in bacteria, an increase in genome size is often linked with an increase in metabolic complexity, allowing bacteria to produce new enzymes that exploit environmental conditions [85]. However, an increase in complexity is linked with a quadratic increase in regulatory proteins associated with transcription and two-component signal transduction systems [86], [87]. In environments such as soil, efficient regulation of enzyme expression, enabling exploitation of scarce yet diverse, complex nutrients can offer a selective advantage, thus lowering the penalty of slow growth, common amongst dominant bacteria in soil environments [88]. Conversely, in the Antarctic soil environment, organic residues are scarce yet labile, with C and N being mineralisable within a relatively short period of time (90 d) under optimal conditions [89]. In the harsh Antarctic soil environment, maintenance of metabolic versatility comes at a higher cost and, more importantly, reproductive efficiency (promoted by smaller genomes) is crucial for survival and growth. Comparative genomic analyses with nine soil-dwelling, temperate *Paenibacillus* spp. have revealed that *P. darwinianus* genomes contained significantly fewer CDSs as compared to the temperate species. A significantly smaller proportion of genes was identified in paralogous clusters in the *P. darwinianus* genome as compared to the temperate *Paenibacillus* genomes (P<0.01). In addition, of the total CDSs identified in the *P. darwinianus* genomes, significantly fewer CDSs were assigned to COG category, transcription [K] (P<0.01). Finally, *P. darwinianus* strain Br^T^ showed limited catabolic activity [26], indicative of lowered metabolic complexity. It should be noted that although smaller genomes offer a metabolic advantage during reproduction, striking a balance between minimum cellular-doubling time and the ability to respond to, or exploit, changing environmental conditions is also crucial.

The *P. darwinianus* genomes contained several features that were also identified in genomes of cold-adapted bacteria and archaea. These included genes for signal transduction pathways, sigma factors, membrane transporters, motility and sporulation associated genes and mechanisms to deal with cold shock, oxidative and osmotic stresses, thus suggesting their importance in cold adaptation and survival. However, comparative analyses revealed that a large proportion of these features were also present in genomes of temperate species. This suggests that these physiological traits, while not unique to Antarctic soils, are beneficial for growth and survival in a range of soil environments. Transcriptomic- and proteomic-based studies comparing the expression profiles of *P. darwinianus* strains and temperate *Paenibacillus* spp., at relatively low versus high temperatures may elucidate the exact mechanisms for cold-adaptation in these strains.

In conclusion, fewer CDSs, lower paralogous content and the limited catabolic activity suggest the occurrence of genome content scaling, offering *P. darwinianus* a growth advantage in the Antarctic soil environment. Although this study has provided an insight into the metabolic potential of this species in RSR soils, it draws attention to our limited knowledge about the expression of physiological traits that enable survival under extreme conditions and, more importantly, to the lack of complete prokaryotic genomes from Antarctic soil environments. Complete genomes will not only reveal genes essential for survival in the harsh Antarctic soil environment but also assist with the characterisation of selection processes that affect prokaryotic genomes in this environment.

## Materials and Methods

### Habitat and isolation strategy

Soil samples were collected from three sites: Cape Evans (77°38′S, 166°24′E) on Ross Island, Minna Bluff (78°30′S, 169°E) on the mainland coast and the Britannia Drift, Lake Wellman Region (79°55′16.2″S, 156°55′30.7″E) in south-eastern Darwin Mountains [90], [91]. Field permits for sample collection were issued by New Zealand Ministry of Foreign Affairs and Trade. These samples were irradiated with ^6^°Co γ-rays for 80 h at a dose of 288 Gy/h and plated onto PYGV (Peptone Yeast extract Glucose Vitamin) gellan gum-based solid medium at 15°C for up to two months [92]. Following incubation and purification, three isolates Br^T^, CE1 and MB1 were identified as strains of *P. darwinianus* by 16S rRNA gene sequencing. *P. darwinianus* Br^T^ was deposited at the International Collection of Microorganisms from Plants (ICMP), Landcare Research, New Zealand (ICMP no. 19912) and at DSMZ, Germany (DSM 27245). *P. darwinianus* strains CE1 (ICMP no. 20538) and MB1 (ICMP no. 20539) were also deposited at ICMP.

Temperature range data were obtained for strains Br^T^, CE1 and MB1 by growth on PYGV (Peptone Yeast Glucose Vitamin) medium solidified by gellan gum [92]. All PYGV plates were incubated for 56 days at 0, 5, 10, 15, 18, 20, 25, 37, 40 and 45°C.

### DNA extraction and sequencing

The three Antarctic *Paenibacillus* strains were routinely cultured on PYGV gellan gum plates at 15–18°C. High molecular weight DNA was extracted by a modified CTAB (hexadecyltrimethylammonium) and protein lysis method [93]. Briefly, cells were scraped off PYGV gellan gum plates and re-suspended in 740 µl TE buffer, 20 µl lysozyme (100 mg/ml) and incubated for 10 min at room temperature. Then, 40 µl of 10% SDS and 8 µl of Proteinase K (10 mg/ml) were added and the reaction incubated overnight at 37°C. Following incubation, 100 µl of 5 M NaCl and CTAB/NaCl solutions were added to each reaction and incubated at 65°C for 10 min. Subsequently, 0.5 ml chloroform∶isoamyl alcohol (24∶1) was added, and the reaction was centrifuged at 16,000 g for 15 min. The aqueous phase was transferred to a clean eppendorf tube containing phenol∶chloroform∶isoamyl alcohol (25∶24∶1, pH 8) and centrifuged at 16,000 g for 15 min. The aqueous phase was transferred to a clean eppendorf tube containing 0.6 vol isopropanol. For DNA precipitation, reactions were incubated at room temperature for 60 min, then centrifuged at 16,000 g for 30 min. The DNA pellet was washed with 70% ethanol and re-suspended in TE buffer containing RNAse (99 µl TE buffer +1 µl RNAse (10 mg/ml)) and incubated at 37°C for 20 min. DNA extracts were quantified by Quant-iT PicoGreen dsDNA assay kit (Life Technologies) and their purity (A_260_/A_280_) was assessed on a NanoDrop ND-1000 Spectrophotometer (Biolab). The quality of each DNA extract was tested by electrophoresis on a 1% agarose gel. Following extraction, high molecular weight DNA was sent to Macrogen (Seoul, South Korea) for sequencing on the Illumina HiSeq 2000 platform using 100 bp paired end libraries. With a sequencing output of 35 Gb, estimated coverage was over 1,000× per genome.

### *De novo* assembly, annotation and comparative analyses

FASTQ files obtained for each genome were trimmed by the FASTQ Trimmer tool of the FASTX-toolkit v0.0.13 [94] and quality filtered by Sickle (https://github.com/ucdavis-bioinformatics/sickle). High-quality reads (Q>30) were assembled into contigs by Velvet v1.2.10 [95]. All assemblies were further improved by tools of the PAGIT (post assembly genome improvement toolkit) pipeline. These included IMAGE (iterative mapping and assembly for gap elimination) for gap elimination and iCORN (iterative correction of reference nucleotides) for sequencing error correction [96]. Improved contigs were assembled into scaffolds by SSPACE basic version 1.0 (stand-alone scaffolder of pre-assembled contigs using paired-read data) [97]. Gene prediction and annotation was performed by the automated JGI (Joint Genome Institute) Pipeline [25] and the NCBI Prokaryotic Genome Annotation Pipeline (PGAP) [98]. Genome completeness was assessed as previously described [99].

For comparative analyses, nine temperate, soil-dwelling *Paenibacillus* species, namely *P. curdlanolyticus* YK9, *P. daejeonensis* DSM 15491, *P. mucilaginosus* K02, *P. panacisoli* DSM 21345, *P. pinihumi* DSM 23905, *P. polymyxa* SC2, *P. taiwanensis* DSM 18679, *P. terrae* HPL-003, and *P. terrigena* DSM 21567 were selected [100]. All analyses were performed using JGI-IMG/ER (Integrated Microbial Genomes/Expert Review) [25] and CMG biotools [43].

### Genome Submission

This whole genome shotgun project has been deposited at DDBJ/EMBL/GenBank under the following accession numbers: JFHT00000000 for strain Br, JFHU00000000 for strain CE1, and JFHV00000000 for MB1.

## Supporting Information

Figure S1 Dot plot comparisons of the three *P. darwinianus* strains.
(PPTX)

Table S1 List of Pfam domains associated with Two-Component Systems in genomes of temperate *Paenibacillus* spp. and *P. darwinianus* strains.
(XLSX)

Table S2 List of genes associated with Two-Component Systems in genomes of temperate *Paenibacillus* spp. and *P. darwinianus* strains.
(XLSX)

Table S3 List of sigma factors identified in genomes of the *P. darwinianus* strains.
(XLSX)

Table S4 List of genes associated with transporter proteins in genomes of temperate *Paenibacillus* spp. and *P. darwinianus* strains.
(XLSX)

Table S5 List of genes associated with ABC-type transport systems in genomes of temperate *Paenibacillus* spp. and *P. darwinianus* strains.
(XLSX)

Table S6 List of COGs associated with carbohydrate metabolism in genomes of temperate *Paenibacillus* spp. and *P. darwinianus* strains.
(XLSX)

Table S7 Sporulation associated genes identified in genomes of the *P. darwinianus* strains.
(XLSX)

Table S8 Sporulation associated genes identified in genomes of temperate *Paenibacillus* spp.
(XLSX)

Table S9 List of flagellar assembly and chemotaxis associated genes in genomes of temperate *Paenibacillus* spp. and *P. darwinianus* strains.
(XLSX)

Table S10 List of genes associated with oxidative and osmotic stress response in genomes of temperate *Paenibacillus* spp. and *P. darwinianus* strains.
(XLSX)

Table S11 List of cold-shock induced genes identified in genomes of temperate *Paenibacillus* spp. and *P. darwinianus* strains.
(XLSX)
