# Supplementary material for: Genome-Based Comparative Analyses of Antarctic and Temperate Species of Paenibacillus
Source: PLoS One. 2014 Oct 6;9(10):e108009. doi: 10.1371/journal.pone.0108009 (PMC4186907; doi:10.1371/journal.pone.0108009)
Supplement: Figure S1 — Dot plot comparisons of the three P. darwinianus strains. (PPTX) [file pone.0108009.s001.pptx]

## Slide 1
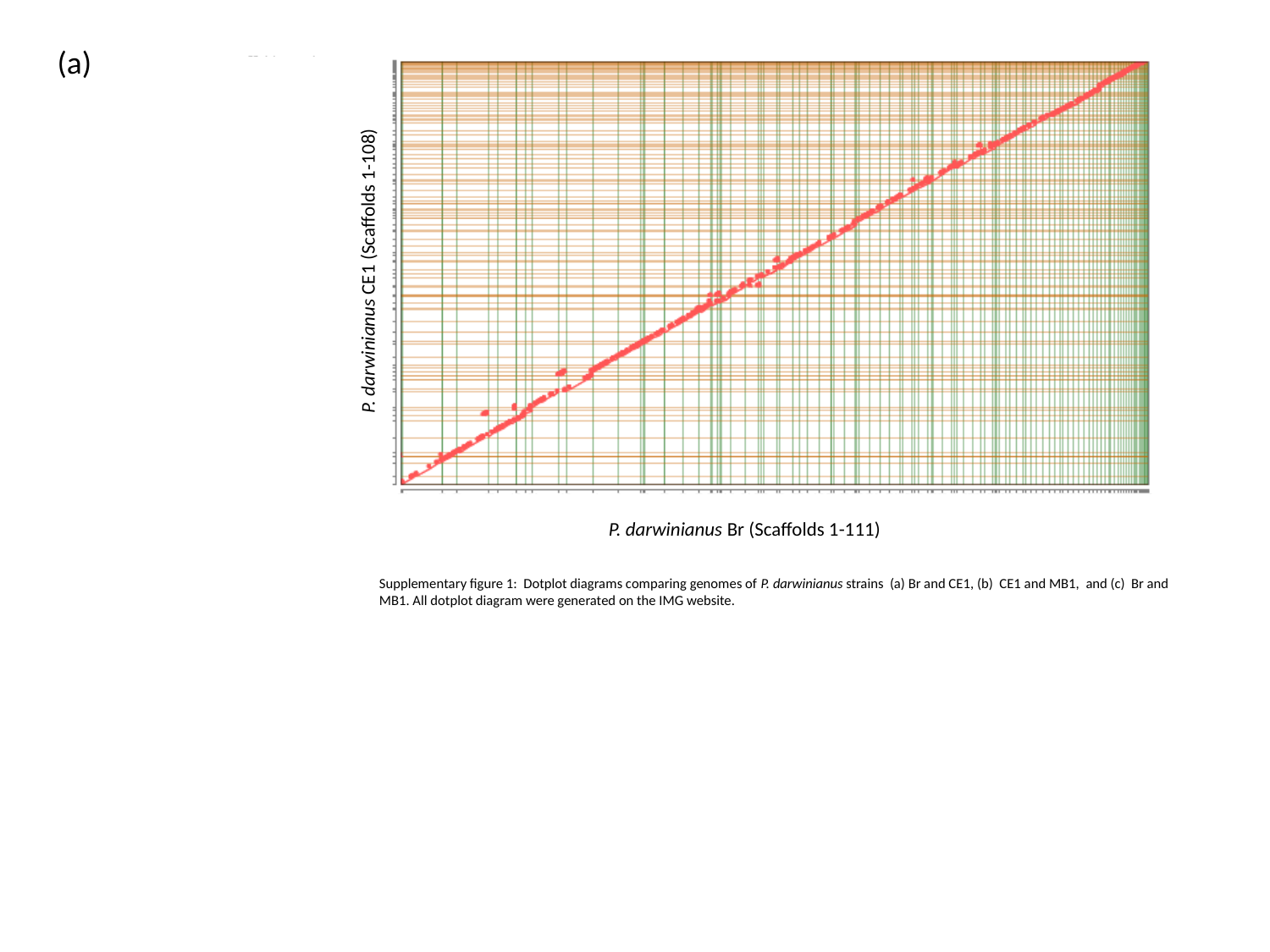

(a)
P. darwinianus CE1 (Scaffolds 1-108)
P. darwinianus Br (Scaffolds 1-111)
Supplementary figure 1: Dotplot diagrams comparing genomes of P. darwinianus strains (a) Br and CE1, (b) CE1 and MB1, and (c) Br and MB1. All dotplot diagram were generated on the IMG website.

## Slide 2
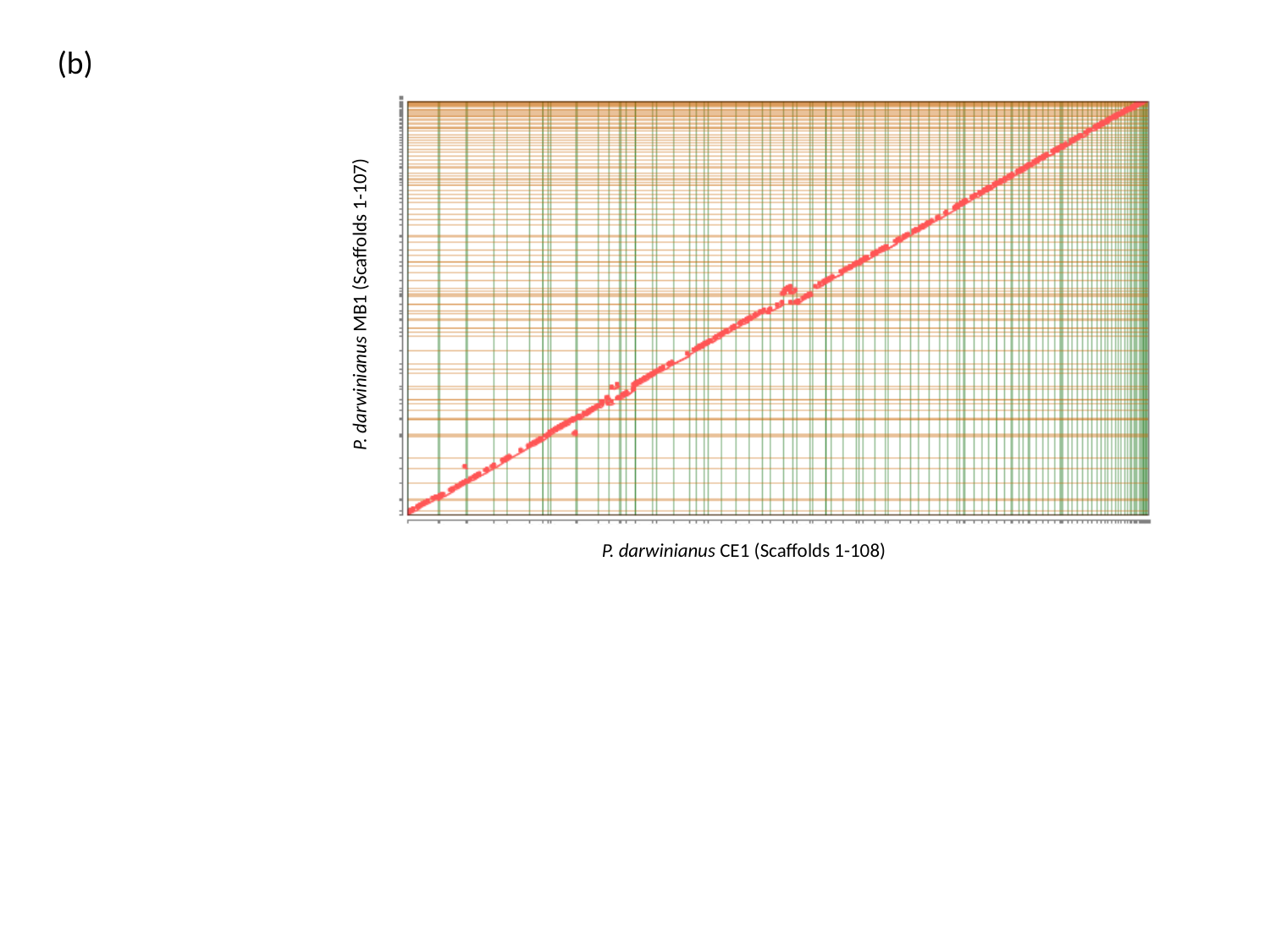

(b)
P. darwinianus MB1 (Scaffolds 1-107)
P. darwinianus CE1 (Scaffolds 1-108)

## Slide 3
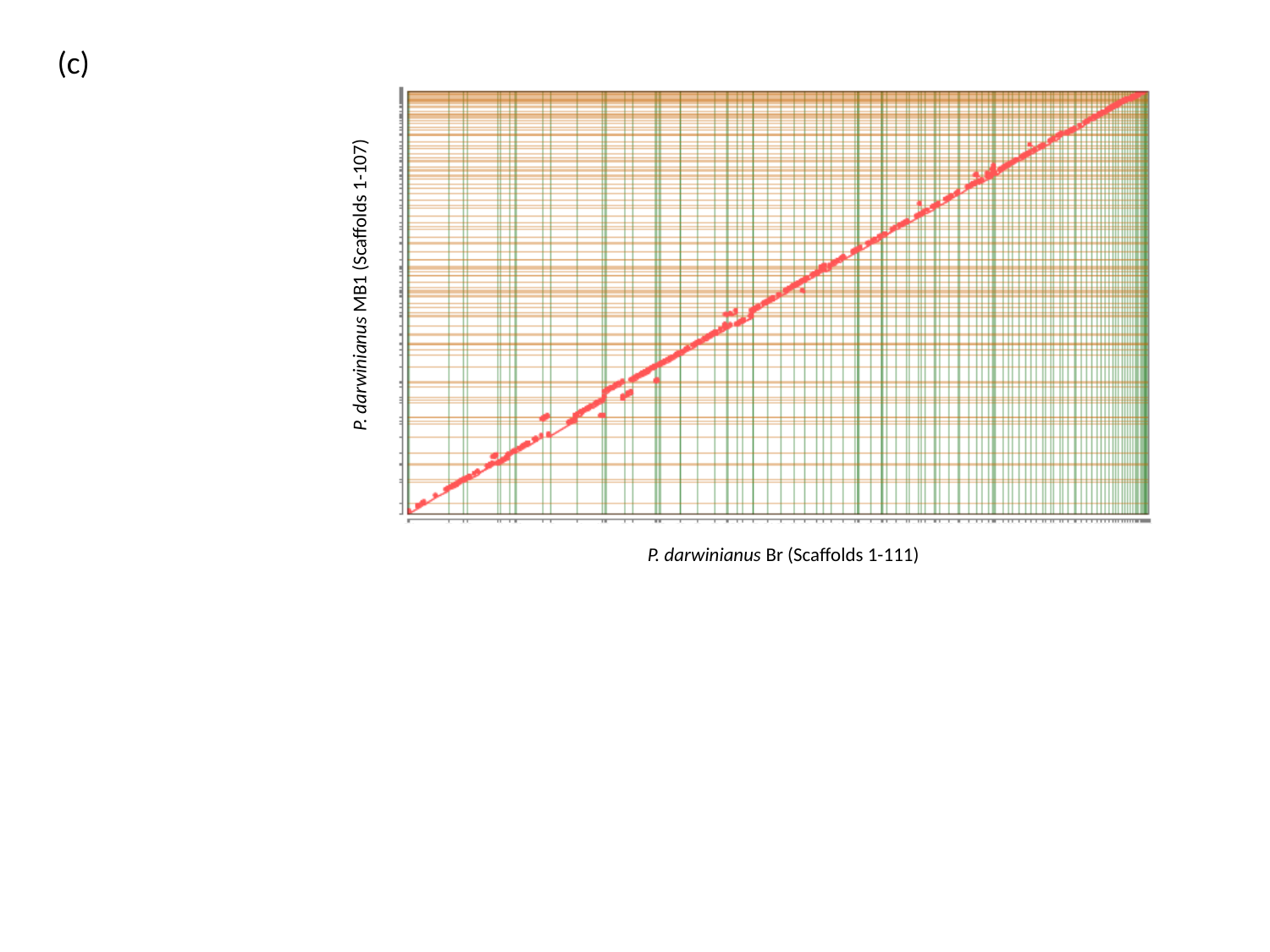

(c)
P. darwinianus MB1 (Scaffolds 1-107)
P. darwinianus Br (Scaffolds 1-111)
